# Whole-Genome Sequence of the Purple Photosynthetic Bacterium *Rhodovulum sulfidophilum* Strain W4

**DOI:** 10.1128/genomeA.00577-13

**Published:** 2013-08-08

**Authors:** Shinji Masuda, Koichi Hori, Fumito Maruyama, Shukun Ren, Saori Sugimoto, Nozomi Yamamoto, Hiroshi Mori, Takuji Yamada, Shusei Sato, Satoshi Tabata, Hiroyuki Ohta, Ken Kurokawa

**Affiliations:** Center for Biological Resources and Informatics, Tokyo Institute of Technology, Yokohama, Japana; Earth-Life Science Institute, Tokyo Institute of Technology, Tokyo, Japanb; Section of Microbial Genomics and Ecology, Tokyo Medical and Dental University, Tokyo, Japanc; Graduate School of Bioscience and Biotechnology, Tokyo Institute of Technology, Yokohama, Japand; Kazusa DNA Research Institute, Chiba, Japane

## Abstract

We report the draft genome sequence of the purple photosynthetic bacterium *Rhodovulum sulfidophilum*. The photosynthesis gene cluster comprises two segments—a unique feature among photosynthesis gene clusters of purple bacteria. The genome information will be useful for further analysis of bacterial photosynthesis.

## GENOME ANNOUNCEMENT

*Rhodovulum sulfidophilum* is a marine photosynthetic purple bacterium (a member of the α3 subclass of proteobacteria) that can grow by either photosynthesis or respiration using a wide range of organic compounds (1, 2). *R. sulfidophilum* has been used as a model to study mechanisms underlying control of photosynthesis gene expression because, unlike most purple bacteria, the synthesis of photosystem components is not repressed or is only slightly repressed under aerobic conditions (3–5). This bacterium is also capable of oxidizing sulfide or thiosulfate to yield sulfates without accumulating intermediates (1, 6) and to have an unusual tri-heme cytochrome subunit bound to the reaction center (7). To better understand its photosynthesis, sulfide oxidation, and regulation of photosystem synthesis, we determined the genome sequence of the *R. sulfidophilum* strain W4 (DSM 1374) (1).

Genome sequencing was initially performed using the Roche/454 GS FLX titanium instrument. Sequencing yielded 344,922,823 bp of DNA, 429,298 reads of ~400 bp in length, and 676,408 mate-pair reads with an average insert length of 8 kbp. Genome sequencing was also performed with Illumina-GAIIx, which yielded 197,091,650 bp of DNA with 2,672,156 paired-end reads of 75 bp. Assembly was performed using Newbler 2.6. The draft assembly comprises nine contigs at 50-fold coverage, two of which are self-circularized and predicted to be plasmids—plasmid 1 (113,621 bp) and plasmid 2 (102,113 bp). The chromosome assembly comprises a single 4,130,470-bp scaffold consisting of seven contigs. The calculated GC content of the draft genome is 66.8%. Gene prediction and functional annotation were carried out with the Microbial Genome Annotation Pipeline (MiGAP) (http://www.migap.org/index.php/en) (8), revealing 3,983 protein-cording genes on the chromosome, 93 on plasmid 1, and 84 on plasmid 2. We also found 50 tRNA genes and 3 rRNA operons on the chromosome.

Most photosynthetic genes, including those involved in bacteriochlorophyll and carotenoid synthesis or encoding light-harvesting apo-proteins and reaction center components, were clustered on the genome to form the so-called photosynthesis gene clusters (PGCs) (9). Interestingly, PGCs in *R. sulfidophilum* comprise two segments separated by ~300 kbp; one includes *pufQBALMC-bchCXYZ-crtEFDC*, and the other includes *crtBIA-bchIDO-puhABCE-bchMLHBNFEJGP*. This existence of two distinct PGCs is unique among purple bacteria. We identified five photosynthetic regulatory genes: *regB*, *regA*, *aerR/ppa*, *ppsR*, and *fnr*. Interestingly, plasmid 1 contains a gene encoding a BLUF domain-containing protein (10). The deduced amino acid sequence of the full-length BLUF protein revealed ~22% identity to *Rhodobacter sphaeroides* AppA, which functions as a blue-light receptor controlling photosynthesis gene expression (11, 12). This suggests the existence of blue-light-dependent regulation of photosystem synthesis in *R. sulfidophilum*. This is the first identification of an AppA ortholog in a species other than *R. sphaeroides*. The *R. sulfidophilum* genome presented here will contribute to our understanding of PGC evolution, the regulation of photosynthesis in purple bacteria, and BLUF photoreceptor-mediated signal transduction.

### Nucleotide sequence accession numbers.

This *R. sulfidophilum* DSM 1374 genome sequence has been deposited at DDBJ/EMBL/GenBank under the accession numbers BASI01000001 to BASI01000009, DF260912 to DF260914 (scaffolds), and PRJDB1308 (BioProject).
